# Methods for the Drug Effectiveness Review Project

**DOI:** 10.1186/1471-2288-12-140

**Published:** 2012-09-12

**Authors:** Marian S McDonagh, Daniel E Jonas, Gerald Gartlehner, Alison Little, Kim Peterson, Susan Carson, Mark Gibson, Mark Helfand

**Affiliations:** 1 Department of Medical Informatics and Clinical Epidemiology and Evidence-based Practice Center, School of Medicine, Oregon Health & Science University, Portland, OR, USA; 2 Department of Medicine, University of North Carolina, Chapel Hill, NC, USA; 3 Cecil G. Sheps Center for Health Services Research, Chapel Hill, NC, USA; 4 International, Research Triangle Park, NC, USA; 5 Oregon Health & Science University Center for Evidence Based Policy, Portland, OR, USA; 6 Veteran’s Affairs Medical Center and Evidence-based Practice Center, Portland, OR, USA; 7 Danube University, Krems, Austria

## Abstract

The Drug Effectiveness Review Project was initiated in 2003 in response to dramatic increases in the cost of pharmaceuticals, which lessened the purchasing power of state Medicaid budgets. A collaborative group of state Medicaid agencies and other organizations formed to commission high-quality comparative effectiveness reviews to inform evidence-based decisions about drugs that would be available to Medicaid recipients. The Project is coordinated by the Center for Evidence-based Policy (CEbP) at Oregon Health & Science University (OHSU), and the systematic reviews are undertaken by the Evidence-based Practice Centers (EPCs) at OHSU and at the University of North Carolina. The reviews adhere to high standards for comparative effectiveness reviews. Because the investigators have direct, regular communication with policy-makers, the reports have direct impact on policy and decision-making, unlike many systematic reviews. The Project was an innovator of methods to involve stakeholders and continues to develop its methods in conducting reviews that are highly relevant to policy-makers. The methods used for selecting topics, developing key questions, searching, determining eligibility of studies, assessing study quality, conducting qualitative and quantitative syntheses, rating the strength of evidence, and summarizing findings are described. In addition, our on-going interactions with the policy-makers that use the reports are described.

## Background

Created from efforts to improve formulary policy for the Oregon Health Plan (OHP) – Oregon’s innovative Medicaid program – the Drug Effectiveness Review Project (DERP) now has almost a decade of experience conducting comparative effectiveness research to determine what works best in the effort to inform health policy decisions with evidence. Recent health care legislation and, in particular, the creation of the Patient-Centered Outcomes Research Institute (PCORI) make this experience increasingly relevant to the national effort to make better and more cost-effective health policy.

By 2001, a rapid increase in the cost of medications [1] had contributed to skyrocketing budgets for the expanded OHP. Governor John Kitzhaber, an emergency room physician by training and a creator of the OHP, believed the application of an evidence-based process would improve health and financial outcomes. Kitzhaber argued that doctors don't have the necessary, unbiased drug comparison studies to help them make choices that are both medically sound and cost effective. These choices should be based on true comparative effectiveness evidence. Cost would be considered primarily where no clinically important differences in benefit or harm were found.

At the outset, the Oregon Evidence-based Practice Center (EPC) was asked to conduct systematic reviews of several classes of drugs; each class contained multiple expensive drugs and thus a potential for increased benefit, reduced harm, reduced cost, or a combination of these. EPC investigators met with the relevant State appointed topic-specific committees (comprising of clinicians and community representatives) initially to develop the key questions and later to provide an overview of the findings and to answer questions. These meetings were held publicly and public testimony was allowed; typically a few physicians, patients (or caregivers), and representatives of pharmaceutical companies attended. The committees made recommendations to the Health Resources Commission, who in turn, made final recommendations to the state Medicaid agency.

Oregon reaped early success with this process: In 2001 a systematic review showed that among the five proton pump inhibitors available, there were no clear differences in benefits or harms between medications [2]. The State ultimately listed only the three least expensive drugs as “preferred.” On the basis of this and other early successes, state Medicaid agencies in Idaho and Washington joined Oregon to commission further research.

**Figure 1:**
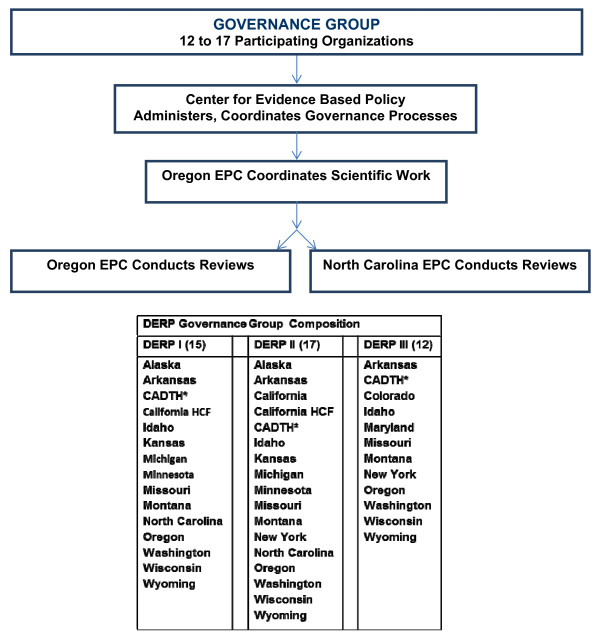
DERP organizational structure.

After leaving office in 2003, Kitzhaber formed the Center for Evidence-based Policy (CEbP) at Oregon Health and Science University (OHSU). The first CEbP project was an expansion of the alliance of Medicaid agencies. In all, 15 organizations – 14 state Medicaid agencies and the Canadian Office for Health Technology Assessment (now the Canadian Agency for Drugs and Technologies in Health) – formed the collaboration that became the DERP (Figure 1). Currently, EPC researchers from OHSU and the University of North Carolina provide comparative effectiveness reviews for DERP.

Over its lifespan, DERP has evolved methodologies to evaluate comparative evidence on the balance of drug therapy benefits and harms and to disseminate systematic reviews of that evidence in collaboration with a highly engaged stakeholder group. DERP’s regular interaction with policy-makers who use the reviews, its experience in determining when topics require updating, and its incorporation of emerging methodological standards, places DERP in a unique position to inform the recent initiatives in comparative effectiveness research. The purpose of this paper is to give an overview of the current methods of evidence review and dissemination used by the two EPCs producing DERP reports.

### Overview of DERP systematic review process

**Figure 2:**
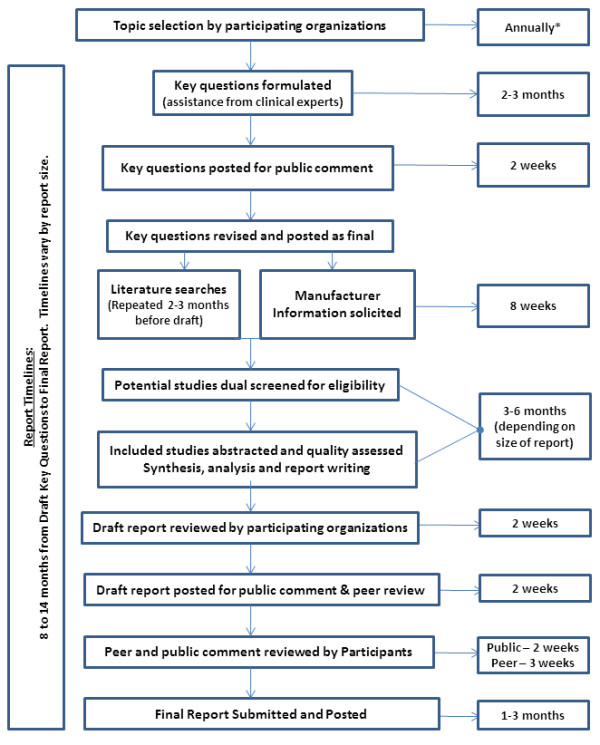
**DERP report process and timelines.** *Depending on funds remaining.

Below is a description of the processes used in producing DERP reports. The overall process and timelines are shown in Figure 2.

#### Conflict of interest policy

DERP investigators and their staff comply with a policy on conflicts of interest that includes a formal, annual, written self-declaration of no financial interest (defined as direct ownership of stock, research funding, or fees for speaker’s bureaus or consulting) in any pharmaceutical company for at least the duration of the time the person is doing work for DERP. We have found that this policy, which is much more restrictive than those at other organizations where modest interests are allowed with disclosure, both simplifies interactions with teams and reassures the stakeholders. At the same time, engagement with industry is a critical part of the DERP process as described below.

#### Selection of topics

Organizations participating in DERP follow an explicit process to ensure equal opportunity for input in the selection of topics for systematic review. The CEbP solicits topics from each organization, typically on an annual basis. After discussing each nomination, the organizations select up to five topics for further workup. Limiting to five topics reflects the workload required to prepare topic briefs, the limited resources available for conducting research, and the improved level of discussion with fewer choices. Consisting of the original submission details, a summary of pros and cons, and an overview of available systematic reviews and randomized trials, this workup is used to inform organizations as to whether sufficient evidence is available to support a full review. Over the years, the content of these briefing papers has evolved. For example, previous versions asked participating organizations to estimate the level of use and potential economic impact of the proposed drugs in their respective programs. Because this was not always possible to do, and created additional workload, this aspect was changed to a more qualitative question about pros and cons. To identify high-quality systematic reviews relevant to the proposed topic, the EPC investigators search MEDLINE and the Web sites for Agency for Healthcare Research and Quality (AHRQ), the Canadian Agency for Drugs and Technologies in Health, the Cochrane Collaboration, and the National Coordinating Center for Health Technology Assessment, the National Institute for Clinical Excellence, and the Center for Reviews and Dissemination. Identification of an existing recent high-quality review may indicate that it is not necessary to undertake a new review and the number of trials is an indicator of the volume of the evidence on a topic. At a face-to-face meeting, participating organizations discuss the briefing papers and select topics for which DERP reports will be commissioned.

#### Formulation of key questions

Key questions define the scope of a DERP report. Preliminary key questions are formulated by EPC investigators on the basis of the discussion that led to the topic selection. Participating organizations review the draft key questions and clinical experts, described below, are consulted. After modifications, the draft key questions are posted to the DERP Web site for public comment. Public comments and responses proposed by investigators are discussed with the participating organizations. Our records indicate that we receive a mean of 2.5 sets of comments per posting.

**Figure 3:**
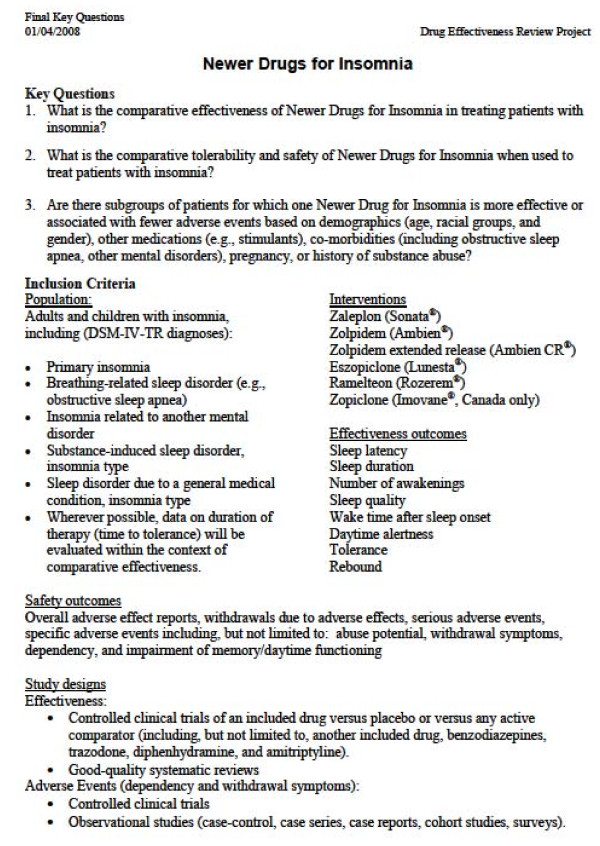
Final key questions and eligibility criteria.

After further modifications, approved by the participating organizations, the final key questions and related eligibility criteria, which will define the scope of the upcoming report, are posted to the DERP Web site. The eligibility criteria specify the Populations, Interventions, Comparisons, Outcomes, Timing, Settings and Study designs or characteristics (PICOTS) of interest. Each of the PICOTS is selected to reflect participant needs. For example, the list of interventions is selected to reflect the drugs under consideration by participating Medicaid agencies and similar drugs available in Canada. In DERP, the primary outcomes of interest are health outcomes reflecting effectiveness rather than short-term efficacy outcomes (e.g., intermediate or surrogate outcomes). However, there are examples where intermediate outcomes are valuable to policy-making and these are included on a case-by-case basis (e.g., LDLc for statins). Eligible harms outcomes typically include overall rates of adverse events (AEs), withdrawal due to AEs, and AEs specific to the drug class, including serious AEs. An example of a set of final key questions and eligibility criteria are shown in Figure 3.

#### Input of clinical advisors

The Clinical Advisory Group provides clinically relevant counsel throughout the development of a report. We began using such an advisory group in 2007, and they are convened for all original reports on new topics. For updates of existing DERP reports, advisory groups are formed on a case-by-case basis, depending largely on whether a substantial change in scope has occurred between the previous report and the pending update. Prior to 2007, we consulted with clinicians on an ad hoc basis.

The CEbP identifies potential clinical advisors among clinicians suggested by the participating organizations, which recommend clinical experts who best represent their constituencies and who also have significant recent experience in direct patient care. The participating organizations review the qualifications and declared conflicts of interest of the group prior to selecting the group’s final composition. While there are no rules on exclusion based on conflicts of interest, if conflicts are declared members are selected with these in mind, in an effort to compile a group with balanced interests. Members of the Clinical Advisory Group are not compensated for their time, typically two to eight hours each, excluding time spent reviewing the draft report if they serve as a peer reviewer. Their names are listed both in the report and on the DERP Web site.

#### Searching for literature

Searches of electronic databases are conducted in consultation with an information specialist. All DERP reports entail, at minimum, a search of Ovid Medline – or PubMed – and the Cochrane Library. Other databases (e.g., Embase and PsycINFO) are searched depending on availability to the EPC conducting the review and on the topic of the report (e.g. PsychINFO is searched for topics that include psychiatric indications). Search strategies generally combine all included interventions (using proprietary and generic names) and populations. Literature searches are repeated 2 to 3 months before submission of the draft report. Additionally, references of key papers are searched by hand.

#### Pharmaceutical companies

The CEbP requests information from all pharmaceutical companies that manufacture a drug included in a DERP report. We request a complete list of citations for all relevant studies of which the manufacturer is aware. We also request information on unpublished studies or unpublished data relating to a published study, with the understanding that once the report is published the public may obtain the information by requesting a copy of the information submitted. An accounting of companies providing information is included in the report. We have received over 100 submissions since DERP began.

#### US Food and Drug Administration (FDA)

The FDA Center for Drug Evaluation and Research “Drugs@FDA” Web site is searched for statistical and medical reviews written by FDA personnel about drugs approved for specific indications. These reviews contain information about trials submitted by pharmaceutical companies as part of New Drug Applications, including study design, results, and analysis of results. We compare the studies submitted to the FDA with those found in the published literature to identify additional unpublished studies or data, including identifying variation in outcomes reporting. FDA documents may not be available for every drug included in DERP reports: posting of a new drug’s documents to the Web site has sometimes been delayed despite approval of the drug and the Web site does not generally contain documents related to drugs approved before 1997.

#### Eligible study designs

For a study to be selected for inclusion, it must meet all eligibility criteria, as described above, including study designs, as explicitly specified *a priori*. Studies with results presented only in a language other than English are excluded because of resource limitations. Studies reported only in conference abstracts are excluded because experience has shown us that typically there is inadequate information provided to evaluate study quality, including selective reporting of results, and to match up multiple abstracts related to a single unique study.

#### Head-to-head trials

Randomized controlled trials directly comparing two or more drugs in the review are included to evaluate both benefit and harms outcomes.

#### Observational studies

Our methods for decisions on including observational studies concur with the recently published guidance for the AHRQ Effective Healthcare Program [3,4]. Head-to-head cohort studies and case-control studies are included in all DERP reports to assess harms [3-6]. These types of studies are also included to evaluate benefits where trial evidence flaws are known or suspected (e.g., trial evidence includes such narrowly defined populations, outcome measures, or interventions and controls that it has limited usefulness). In our reports on drugs to treat attention deficit hyperactivity disorder (ADHD), we made the decision to include observational studies for benefit outcomes because the trials 1) were largely conducted in controlled classroom settings, 2) enrolled children with few or no comorbidities, and 3) did not report “real life” outcomes, such as academic progress or success [7]. While *all* DERP reviews include observational studies to assess harms, to date seven also include them to assess benefits.

#### Systematic reviews

Existing systematic reviews that *directly* address the key questions and meet minimum standards for quality are considered for inclusion. Reviews must meet the following minimum criteria to be considered systematic: The review must include a comprehensive search for evidence from multiple sources of information (electronic databases, reference lists, etc.), describe the terms used in the search (in some way), and use dual review of studies for inclusion. The review must address questions that are similar enough to the key question to provide useful information; reviews that examine a class effect or only a portion of the drugs in a class are unlikely to be useful in a DERP report. For example, while we identified many prior systematic reviews of atypical antipsychotic drugs, none made comparisons of all drugs in the group to each other [8]. Most compared only olanzapine and risperidone, or compared the atypical drugs to the older, conventional antipsychotic drugs. A cut-off date is selected (e.g., two years) based on how much the field has changed in the intervening period. With atypical antipsychotics, the reviews identified were outdated and new studies and new drugs needed to be considered.

#### Placebo-controlled trials

Where there are gaps in direct comparative evidence, we consider including placebo-controlled trials for indirect comparisons of benefits and harms. The decision to pursue a quantitative indirect comparison takes into account the degree of clinical, methodological, and statistical heterogeneity across the studies under consideration. We follow established guidance on methods for quantitative indirect synthesis [3,9-14].

When indirect comparisons are not undertaken, findings from placebo-controlled trials can be discussed to identify individual drugs with evidence of benefit (and to identify the magnitude of effect) and those with no such evidence.

#### Study-level pooled analyses

“Pooled analyses” is our term for published meta-analyses that are not based on a comprehensive literature search and do not include assessment of the quality of individual studies. Pooled analyses differ from systematic reviews that utilize meta-analysis as part of the synthesis of evidence (e.g. DERP reports) and potentially introduce bias. Therefore, as with placebo-controlled trials, pooled analyses are considered for inclusion in DERP reports only where other evidence is absent or insufficient. An example is where the only information about a subgroup of patients available is from a pooled analysis of data from manufacturer-sponsored trials.

#### Single-group studies

Studies without a well-formed comparison group have a high risk of bias, particularly in assessing benefit [4]. Single-group studies may be included to evaluate harms under the “best evidence” approach only if the studies add important evidence on harms that is not available from study designs with lower risk of bias. To be useful a single-group study should have both adequate sample size and duration of drug exposure that is both longer than the exposure in existing trials (e.g. where trials range from a few weeks to several months but single-group studies report a year or more of exposure) and long enough for AEs that take time to develop to occur (e.g. cancer). We often depend on the clinical experience of our advisors to determine the required length of observation required for a given AE, because it can be difficult to make an objective determination. Such studies may have included patients with a broader range of comorbidities, increasing applicability.

Open-label extension studies have limited applicability and higher risk of bias than do the original trials. These study populations are derived from clinical trials, where patients are already winnowed to meet a narrow set of eligibility criteria, and patients in the extension often are those who had an adequate drug response and tolerance during the trial period. An extension study without a comparison group is especially subject to bias, becoming essentially an observational study in a highly selected population. As such, we include them only under the conditions stated above, but view them as reflecting a very narrowly defined population.

#### Unpublished studies or data

Unpublished studies (e.g. information submitted by manufacturers, FDA documents, or trial registries that include results) may be included if they meet eligibility criteria and provide sufficient detail to assess study quality. At minimum, information must be provided on the comparability of groups at baseline, attrition, number of patients analyzed, the statistical tests used for data analysis, and whether an intention-to-treat analysis was conducted. When a manufacturer provides additional unpublished outcomes or subgroup data from a published study, these data will be included if 1) the study is a direct, head-to-head comparison of included drugs, and 2) the study reports the statistical tests used for data analysis, attrition, numbers of patients analyzed in each group, and whether an intention-to-treat analysis was conducted. Prior to 2010, inclusion criteria were determined on a case-by-case basis by each review team, largely based on the ability to conduct a quality assessment. Experience such as the limited usefulness of including various post-hoc subgroup data submitted by industry for our fixed dose combination products review led us to tighten and formalize these criteria in 2010 [15].

#### Determining eligibility of studies

In order to reduce potential reviewer bias and ensure accuracy and reproducibility, all study reports identified in literature searches are assessed for eligibility by two investigators. Before full-text studies are assessed, titles and abstracts identified by searches are evaluated. For this evaluation one investigator may complete eligibility assessments with a second reviewer evaluating only those studies that the first investigator chose to exclude. With this “carry-forward” strategy, only studies that are unequivocally ineligible are rejected at this stage. Full-text articles of potentially relevant citations are retrieved and dually assessed for inclusion by two reviewers. Disagreements are resolved through consensus. In accordance with recommendations on reporting, a diagram indicating the flow of inclusion and exclusion of studies is presented [16]. Excluded trials, along with the reason for exclusion, are listed in an appendix.

#### Data abstraction

Data routinely abstracted from studies are: study design, population characteristics, eligibility criteria, interventions, numbers randomized/treated and analyzed, and results. Abstraction is performed by one reviewer and independently checked by another; differences are resolved by consensus. We record intention-to-treat results when reported. If not reported, but loss to follow-up was very small (< 5%), we note that they were modified intention-to-treat results.

#### Quality assessment of individual studies

Historically, to assess quality (i.e., internal validity or risk of bias) of trials, cohort studies, and case-control studies, we have applied predefined criteria that are based on those used by the US Preventive Services Task Force and the (UK) National Health Service Centre for Reviews and Dissemination [17,18]. Studies that have a fatal flaw are rated poor quality; studies that meet all criteria are rated good quality; the remainder are rated fair quality. As the fair-quality category is broad, studies with this rating vary in their strengths and weaknesses— the results of some fair-quality studies are likely to be valid, while others are only possibly valid. A poor-quality study is not valid and the results are at least as likely to reflect flaws in the study design as a true difference among the compared drugs. A fatal flaw may be reflected by one aspect introducing a high risk of bias or by failure to meet combinations of items of the quality assessment checklist. An example would be a study with a high attrition (e.g., 60%) combined with inadequate handling of missing data (e.g., analyses based on observed events). A particular study might receive different ratings for different outcomes.

The items assessed for trials, observational studies, and systematic reviews are shown in Table 1. Each study is assessed by two reviewers, with disagreements resolved through consensus. There are currently no established criteria for evaluating pooled analyses or single-group studies.

**Table 1:** Drug Effectiveness Review Project (DERP): Quality assessment criteria checklists by study design

**Study design**	**Controlled clinical trial**	**Observational study**	**Systematic review**
Quality Assessment Criteria	·Randomization adequate?	·Non-biased selection?	·Report clear review question, state inclusion and exclusion criteria of primary studies?
	· Allocation concealment adequate?	·High overall loss to follow-up or differential loss to follow-up?	
	·Groups similar at baseline?		
		·Outcomes pre-specified and defined?	·Substantial effort to find relevant research?
	·Eligibility criteria specified?		
		·Ascertainment techniques adequately described?	·Adequate assessment of validity of included studies?
	·Outcome assessors masked?		
	·Care provider masked?	·Non-biased and adequate ascertainment methods?	·Sufficient detail of individual studies presented?
	·Patient masked?		
	·ITT-analysis?	·Statistical analysis of potential confounders?	
	·Maintenance of comparable groups?		·Primary studies summarized appropriately?
		·Adequate duration of follow-up?	
	·Acceptable levels of crossovers, adherence, and contamination?		
			
	·Overall and between-group attrition acceptable?		
			

Abbreviations: *ITT* intention-to-treat.

The EPC program has recently updated their recommendations on assessing the internal validity of studies [19]. While the revised guidance continues to allow EPCs to choose tools that evaluate the broader concept of quality such as the one we use in DERP, it emphasizes approaches that focus on the “risk of bias” of studies.

#### Evidence synthesis

Studies are stratified by key question and study quality. The best evidence informs the synthesis that will address each key question. Studies that evaluate one drug against another provide direct evidence of comparative benefits and harms and are preferred over indirect comparisons. Similarly, studies of effectiveness and long-term or serious harms outcomes are preferred over studies of efficacy and short-term tolerability. Data from indirect comparisons are used to support direct comparisons and as the primary comparison when no direct comparisons exist. Based on a number of statistical assumptions, indirect comparisons are interpreted with caution; among their defects is a greatly reduced statistical power to detect differences among groups [3,13,20,21]. Mixed treatment comparisons meta-analysis (network meta-analyses) is a relatively new method that allows incorporation of both direct and indirect comparisons in a single analysis and can be useful to allow for inclusion of all potentially relevant data (e.g., from both placebo-controlled and head-to-head trials) [22,23].

Evidence tables report study characteristics, quality ratings, and findings of included studies. Key findings of the review are encapsulated in “summary bullets” (followed by a detailed discussion of the evidence) and in a summary table at the end of the report.

#### Quantitative synthesis

For meta-analysis we follow the AHRQ EPC Methods Guide recommendations [3]. In brief, to determine whether meta-analysis can be meaningfully performed, we consider the quality of the studies and the variation in PICOTS. The Q statistic and the I^2^ are calculated to assess statistical heterogeneity among studies and our interpretation of the importance of observed heterogeneity depends on the magnitude and direction of effects and on the strength of evidence for heterogeneity [24-26]. If significant statistical heterogeneity is identified, potential sources can be examined by analysis of subgroups of study design characteristics, study quality, patient population, and variation in interventions, beyond those identified *a priori*. Meta-regression models may be used to formally test for differences among subgroups with respect to outcomes [27,28]. We prefer random-effects models to estimate pooled effects because we believe it provides a more conservative estimate, allowing for some inter-study variation. A fixed effect model is used in cases where inter-study variation is not expected (e.g., two very similar trials). Other analyses, including adjusted indirect meta-analysis and a mixed treatment effect model (network meta-analysis), are considered in consultation with experienced statisticians. To date, eight DERP reports have conducted indirect comparison or network meta-analyses. When synthesizing unpublished evidence, investigators conduct sensitivity analyses where possible to assess any apparent reporting bias. Poor-quality studies are not combined with fair- and good-quality studies in our main analyses, but may be added as part of sensitivity analyses.

#### Grading the strength of the evidence

Strength of evidence is assessed for the main outcomes of each key question, generally by following the approach suggested by the EPC Methods Guide [3]. Using the EPC approach, reviewers assign grades of high, moderate, low, or insufficient to describe their confidence in the strength of the evidence. At minimum, strength of evidence grades take into account the number and severity of limitations in risk of bias, consistency, directness, and precision. Strength of evidence ratings typically focus on a subset of the outcomes that are most important to the priorities of the DERP participants. Representatives of the DERP organizations, and clinical advisors, act as proxies for patients’ views on which health outcomes are most important. Evidence from poor-quality studies does not contribute to the assessment of strength of evidence. The main findings and the strength of the evidence for each key question are summarized in a table at the end of the report, while the individual assessments of strength of evidence for each outcome are included in an appendix. DERP reports have always included an assessment of the quality of the body of evidence; prior to adopting these outcome-based methods, we used the key-question-based methods of the United States Preventive Services Task Force [18]. We found the key-question-based method is simpler to convey to policy-makers, although clinicians appreciate the nuance of the outcome-based method.

#### Applicability

The applicability of the evidence related to each key question is described in terms of populations and interventions to which the evidence applies. Exceptions – groups or interventions to which the evidence does not apply – are specified. Further consideration of how the evidence applies to each organization’s local population is left to the local decision-makers, with EPC investigators available to respond to questions.

#### Peer review and public comment

DERP reports are subject to peer review and public comment prior to finalization. The participating organizations also review the reports and provide comments to the investigators. These processes help to optimize completeness and minimize bias in the final report. Peer reviewers are identified by professional societies, acknowledged expertise in a particular field, prominent authorship in the published literature, or participation as a Clinical Advisory Group member. For two weeks, draft reports are posted to the public DERP Web site for public comment. Manufacturers and groups of individuals with expressed interest in the topic are notified before the posting and a mean of 2.7 sets (range 1–11) were received per draft. The investigators are responsible for documenting a resolution for each comment. The participating organizations are the final arbiters of whether comments have been adequately addressed. Typical comments received are suggestions to add additional studies (a small number that our searches missed but more often studies that do not meet eligibility criteria) and corrections to recorded details of individual studies (e.g., the sample size and the percent in a particular demographic group).

#### Updating reports

The participating organizations consider report updates annually. This decision is based on an EPC-produced scan that identifies new trials, drugs, approved indications, or serious harms relevant to the key questions of a report. The report update process is as for an original report. FDA approval of relevant new drugs is the most common impetus for updating [29]. In the last year, DERP has begun Single Drug Addendums to handle situations where a new drug has become available very soon after a report has been completed. These addendums supplement the complete report until a full update is commissioned and are produced on a 3-month timeline.

#### Interaction with participating organizations

Once a report is produced, participants use them in making evidence-based decisions on drugs in their respective programs and investigators are available upon request to present an overview of findings and to answer questions. The investigators have found these interactions, monthly phone conferences, and biannual meetings to be of great value in identifying and understanding the needs of policy-makers that use DERP reports. Lessons learned through these interactions and from surveys about the organizations’ opinions on various aspects of the project, including the research reports, directly impact future reports. Changes made to the methodology of reports based on these interactions include 1) broadening the scope to include multiple drug classes; 2) the creation of Single Drug Addenda as noted above; 3) creation of “black box warnings” appendices that do not qualify as evidence in the review but are needed by the participants in making decisions; and 4) formatting of summary material (e.g. executive summaries and slides) to fit the needs of the state Medicaid administrators, among others.

## Discussion

DERP’s current evidence review methods were developed primarily to meet the needs of State Medicaid programs for informing evidence-based health policy decisions using public processes. Development of these methods began prior to the existence of the current Methods Guide for EPCs. While many methods are now congruent, there are nuances where DERP methodology is explicit rather than allowing selection from multiple options, largely due to having a specific audience. After 9 years of experience with DERP, we view the advantages of these methods to be:

1. Incorporating input from the participants makes the reports more useful to them for making policy decisions,

2. Encouraging transparency through outside evaluation of work quality and independent assessment for any risk for bias,

3. Inviting public comment at the initial stages and again near the final stages of the review allows for consideration of viewpoints beyond those of academic peer reviewers and the participants of DERP,

4. Using a regular process to scan for new, important developments in a given topic area allows for the best use of participant funds, and

5. Providing specialized summary documents, for example slide sets and executive summaries with new information highlighted to increase the usefulness of the reports for the participants.

While other groups may need to modify the approach depending on the ultimate users of their work, we recommend that organizations producing comparative effectiveness reviews to inform health policy decisions draw on the insights of policy-makers to develop and refine evidence review methods. Our methods may not be directly transferrable to topics other than comparative effectiveness reviews of pharmaceuticals, such as nonpharmacological interventions or assessment of prognostic and diagnostic tools, which may require special methodologic features.

## Conclusions

DERP has developed a timely and efficient method of creating high-quality systematic reviews designed to meet the needs of policy-makers. DERP continues to be an innovator in methods that involve stakeholders. The recent Institute of Medicine report [30] on conducting systematic reviews reflects the DERP experience – recommending direct and continuing communication with policy-makers, flexibility in responding to new situations, and responsiveness to emerging methods in comparative effectiveness reviews. DERP has seen these methods develop over the past 9 years. The result is a series of comparative drug effectiveness reports that have direct impact on policy decisions, reflected by the ongoing financial support of the constituent organizations. Ongoing collaboration across organizations that generate systematic reviews and the policy-makers that use them will be essential to enhancing and disseminating high-quality methods for this work.

## Competing interests

The authors declare that they have no competing interests.

## Authors' contributions

All authors participated in the development of the methods described, with participation over 5 to 9 years. MM led methods discussions and drafted the initial manuscript. DJ, GG, KP, SC, and MH were significant contributors to systematic review methods development over 9 years, and provided comments on the draft manuscript. AL and MG represented the Center for Evidence-based Policy in discussions of methods and contributed significantly to the development of methods for industry relations. All authors read and approved the final manuscript.

## Pre-publication history

The pre-publication history for this paper can be accessed here:

http://www.biomedcentral.com/1471-2288/12/140/prepub
